# Altered expression of protein tyrosine phosphatase, non-receptor type 22 isoforms in systemic lupus erythematosus

**DOI:** 10.1186/ar4440

**Published:** 2014-01-17

**Authors:** Hui-Hsin Chang, William Tseng, Jing Cui, Karen Costenbader, I-Cheng Ho

**Affiliations:** 1 Division of Rheumatology, Immunology, and Allergy, Department of Medicine, Brigham and Women’s Hospital, One Jimmy Fund Way, Boston, MA 02115, USA; 2 Harvard Medical School, 25 Shattuck Street, Boston, MA 02115, USA; 3 Section of Rheumatology, VA Boston Healthcare System, 150 South Huntington Avenue, Boston, MA 02130, USA

## Abstract

**Introduction:**

A C-to-T single nucleotide polymorphism (SNP) located at position 1858 of human protein tyrosine phosphatase, non-receptor type 22 (PTPN22) complementary DNA (cDNA) is associated with an increased risk of systemic lupus erythematosus (SLE). How the overall activity of PTPN22 is regulated and how the expression of PTPN22 differs between healthy individuals and patients with lupus are poorly understood. Our objectives were to identify novel alternatively spliced forms of PTPN22 and to examine the expression of PTPN22 isoforms in healthy donors and patients with lupus.

**Methods:**

Various human PTPN22 isoforms were identified from the GenBank database or amplified directly from human T cells. The expression of these isoforms in primary T cells and macrophages was examined with real-time polymerase chain reaction. The function of the isoforms was determined with luciferase assays. Blood samples were collected from 49 subjects with SLE and 15 healthy controls. Correlation between the level of PTPN22 isoforms in peripheral blood and clinical features of SLE was examined with statistical analyses.

**Results:**

Human PTPN22 was expressed in several isoforms, which differed in their level of expression and subcellular localization. All isoforms except one were functionally interchangeable in regulating NFAT activity. SLE patients expressed higher levels of PTPN22 than healthy individuals and the levels of PTPN22 were negatively correlated with the Systemic Lupus International Collaborating Clinics/American College of Rheumatology Damage Index (SLICC-DI).

**Conclusions:**

The overall activity of PTPN22 is determined by the functional balance among all isoforms. The levels of PTPN22 isoforms in peripheral blood could represent a useful biomarker of SLE.

## Introduction

PTPN22 is a non-receptor type protein tyrosine phosphatase expressed mainly in hematopoietic cells [1]. It contains a basic bipartite nuclear localization signal (NLS) in its N-terminus, which is followed by a conserved protein tyrosine phosphatase (PTP) domain. An inhibitory domain inhibiting its phosphatase activity is located immediately after the PTP domain [2]. Its C-terminal half is relatively less conserved, with the exception of four proline-rich domains. Its physiological function is still not fully understood. PTPN22 has been shown to attenuate the strength of T cell receptor (TCR) signals by interacting with Lck, Csk, and Vav [3-6]. PTPN22-deficient mice developed age-dependent splenomegaly due to hyperactivation of lymphocytes, and knockdown of PTPN22 in human T cells with small interfering RNA (siRNA) led to enhanced TCR-mediated nuclear factor-kappa B (NF-κB) activity [7,8]. PTPN22 is present in both the cytoplasm and nucleus of macrophages [9]. Its nuclear localization requires the NLS proximal to the PTP domain. The expression of PTPN22 is further induced in alternatively activated macrophages through a STAT6-dependent mechanism. Cytoplasmic PTPN22 suppresses the polarization of classically activated macrophages, whereas nuclear PTPN22 promotes the differentiation of alternatively activated macrophages [9].

Alternative splicing is an evolutionary conserved mechanism enabling a cell to produce proteins of different function from a single gene. A large body of evidence has indicated that the process of alternative splicing is correlated with disease activity or is even pathogenic in some autoimmune diseases [10-12]. At least two isoforms of PTPN22 have been reported. Lyp2, of which the sequence was deduced from two complementary DNA (cDNA) fragments, lacks the three most C-terminal proline-rich domains [1], whereas isoform 2, tentatively called PTPN22.2 for the purpose of discussion, splices out exons 10 and 11. However, it is unclear whether Lyp2 and PTPN22.2 are functionally distinct from the full-length PTPN22 (tentatively called PTPN22.1). We have also identified a novel isoform of PTPN22, called PTPN22.6, which lacks nearly the entire PTP domain [13]. Unlike PTPN22.1, overexpression of PTPN22.6 actually increased NFAT-dependent luciferase activity. More importantly, PTPN22.6 can act as a dominant negative mutant of PTPN22.1 in regulating cytokine production in Th cells, suggesting that the overall activity of PTPN22 can be influenced by the relative levels of its isoforms.

Several genome-wide association studies have linked PTPN22 to autoimmune diseases. A C-to-T single nucleotide polymorphism, which is located at position 1858 of PTPN22 cDNA and converts an arginine to a tryptophan, is associated with a higher risk of rheumatoid arthritis (RA), systemic lupus erythematosus (SLE), and type 1 diabetes but reduces the risk of Crohn’s disease [7,14-17]. Despite these observations, it is still unclear whether the expression of PTPN22 in patients with autoimmune diseases differs from that of healthy individuals, and how this would occur. In addition, the functional impact of the C1858T SNP is still controversial and appears to be complicated. The conversion from arginine to tryptophan resulted in both gain and loss of function of PTPN22 in T cells in different studies [18,19]. We also found that the R-to-W conversion in the context of PTPN22.1 resulted in a gain of function of PTPN22 and a reduction of interleukin (IL)-2 production in T cells. In contrast, the R-to-W conversion in the context of PTPN22.6 led to a loss of function of PTPN22 and overproduction of IL-2 [13]. Thus, the functional impact of the C1858T SNP is isoform dependent. Furthermore, the C1858T SNP is associated with a loss-of-function phenotype in resting macrophages but a gain of function in classically activated macrophages [9]. How these C1858T SNP-associated functional changes affect the risk of autoimmune diseases is largely unknown. We have previously shown that the transcript level of PTPN22.6 but not total PTPN22 in peripheral blood is correlated with the 28-joint disease activity score with three variables including C-reactive protein (DAS28-CRP3) scores in patients with RA [13]. However, it is unclear whether the expression of PTPN22 and its isoforms is altered in SLE patients and whether the level of PTPN22 isoforms also is correlated with disease activity of SLE.

Here we report the identification of additional isoforms of human PTPN22. We examined the expression of the PTPN22 isoforms in primary human T cells and macrophages. We further compared the expression of PTPN22 isoforms in the peripheral blood of 15 healthy donors and 49 SLE patients, and correlated the expression pattern of PTPN22 isoforms with clinical features of lupus.

## Methods

### Human samples

Forty-nine individuals with SLE fulfilling the 1997 American College of Rheumatology revised classification criteria [20] were previously recruited from the Lupus Center of the Brigham and Women’s Hospital (BWH) for the Lupus Biobank. Fifteen control samples from healthy individuals without SLE or related connective tissue disease were obtained from the PhenoGenetic project at the same hospital. All study subjects consented to participate in studies conducted through the Lupus Biobank and the PhenoGenetic project, and agreed to the publication of results derived from such studies. Demographics were collected in addition to the following data from all SLE cases: 1) age at SLE onset; 2) SLE manifestations and organ involvement; 3) concurrent anti-double-stranded DNA antibody titer, C3 and C4. The treating rheumatologist performed a history and physical examination for SLE disease activity (Safety on Estrogens in Lupus Erythematosus National Assessment-SLE Disease Activity Index; SELENA-SLEDAI) [21], and history and chart review to complete an SLE organ damage assessment (SLICC-DI) [22]. Whole blood samples in PAX gene tubes (Qiagen, Germantown, MD, USA) were obtained from all subjects by standard antecubital venipuncture and stored at -80C at the Broad Institute of MIT and Harvard as separated whole blood or extracted RNA. All aspects of the study were approved by the partners’ Institutional Review Board.

### Preparation of human peripheral blood mononuclear cells (PBMC) and helper T (Th) cells

PBMC were isolated from buffy coats. Th cells were enriched from PBMC with CD4 Microbeads (120-000-440, Miltenyi Biotec, Auburn, CA, USA). Macrophages were prepared from PBMC according to a published protocol [23].

### Cell culture and medium

Human primary Th cells and Jurkat cells were cultivated in RPMI-1640. In some experiments, Th and Jurkat cells were stimulated with 2.5 μg/ml of plate-bound anti-CD3 (Hit3a, Cat. # 300314, BioLegend, San Diego, CA, USA) and 2 μg/ml of soluble anti-CD28 (CD28.2, Cat. # 302914, BioLegend) in the presence of IL-2 (50 unit/ml) for an indicated amount of time. Human colonic adenocarcinoma cell line HT-29 cells (HTB38, ATCC) and human embryonic kidney cells 293 T were cultivated in McCoy’s 5A medium (Gibco, Grand Island, NY, USA) and Dulbecco’s modified Eagle’s medium (DMEM), respectively. Primary human macrophages were stimulated with lipopolysaccharide (LPS) (1 μg/ml, InvivoGen, San Diego, CA, USA) along with human interferon-gamma (hIFN-γ) (50 ng/ml, PeproTech, Rocky Hill, NJ, USA) for 24 hours for M1 polarization or hIL-4 (25 ng/ml, PeproTech) along with hIL-13 (25 ng/ml, PeproTech) for 24 hours for M2 polarization.

### Western blotting

Whole-cell extract was obtained by lysing cells with lysis buffer (50 mM Tris–HCl, pH 7.5, 150 mM NaCl, 1% TritonX-100, 0.5% DOC, 0.1% SDS, and 1 mM EDTA) containing 0.5 mM PMSF and complete protease inhibitor cocktail (Roche, Basel, Switzerland). Cytoplasmic and nuclear extracts were prepared by washing cells with cold phosphate- buffered saline (PBS) and resuspending them in hypotonic lysis buffer (10 mM HEPES, pH7.9, 1 mM MgCl2, 10 mM KCl, 0.1% TritonX-100, 20% Glycerol, 0.5 mM PMSF, and protease inhibitors) on ice for 10 minutes. The supernatant, corresponding to cytoplasmic fraction, was collected by centrifugation at 12,000 g for 10 minutes. The nuclear pellet was washed with hypotonic lysis buffer and then resuspended in hypertonic lysis buffer (10 mM HEPES, pH7.9, 400 mM NaCl, 1 m EDTA, 0.1% TritonX-100, 20% Glycerol, 1 mM PMSF, and protease inhibitors) and then incubated on ice for 20 minutes. Nuclear extract was collected by centrifugation. Protein extract was analyzed by immunoblot. The following antibodies were used: human PTPN22 antibody AF3428 (R & D Systems, Minneapolis, MN, USA), Hsp90 α/β (sc-7947) and Lamin B (sc-6216) antibody (Santa Cruz Biotechnology, Inc. Santa Cruz, CA, USA), Oct1 (sc-232, Santa Cruz Biotechnology, Inc.), and FLAG antibody (F3165, Sigma-Aldrich, St Louis, MO, USA).

### Plasmid, transfection and luciferase assay

cDNAs encoding PTPN22.1 and PTPN22.8 were amplified directly from Jurkat cells with primers 5′-CGGGATCCTTGCTCTGCAGCATGGACCAAAGA-3′ and 5′-GACGTCGACGCGTTTAAATATTCCAAGTTGGTGGT-3′. cDNA clones BC071670 (PTPN22.2) and BC017785 (PTPN22.7) were purchased from Open Biosystems (Huntsville, AL, USA). cDNA clones AK3030124 (PTPN22.4), AK310698 (PTPN22.5), and AK310570 (PTPN22.6) were obtained from the NITE Biological Resource Center (Chiba, Japan). All cDNA fragments were cloned into an N-terminal FLAG-tag expression vector pCMV-Tag 2B (Stratagene, La Jolla, CA, USA). Transfection of 293 T cells was performed with Effectene Transfection Reagent (Cat. # 301427, Qiagen). Transfection of Jurkat cells was performed with electroporation with a Gene Pulser II (Bio-Rad, Hercules, CA, USA) set at 374 V/1050 μF. In all NFAT luciferase assays, Jurkat cells were transfected with 5 μg of 3xNFAT-Luc, 0.5 μg pTK-Renilla, and 10 μg of pCMV-Tag 2B expression vectors; rested for 48 hours; and then stimulated with anti-CD3 for 6 hours. Luciferase activity was determined with a Dual-Luciferase™ Reporter Assay System (Promega, Madison, WI, USA). Firefly luciferase activity was then normalized against Renilla luciferase activity obtained from the same sample. 3xNFAT-Luc and pTK-Renilla lucifease vectors were described previously [24].

### Real-time PCR and non-quantitative PCR

Total RNA was prepared using a Trizol Plus kit (Invitrogen, Carlsbad, CA, USA). Reverse transcription was performed on 1 ug of total RNA using the QuantiTect Reverse Transcription kit (Qiagen). Real-time PCR analysis was performed using the Brilliant SYBR Green QPCR kit according to the manufacturer’s protocol (Stratagene) on a MX-3000P apparatus (Stratagene). The cycling conditions are: one cycle of 95C for 10 minutes and 40 cycles of 95C for 30 seconds, 56C for 1 minute, and 72C for 1 minute. The following primer pairs were used: ‘total’ 5′-GCAGAAGTTCCTGGATGAG-3′ and 5′-TCAGCCACAGTTGTAGGATAG-3′; Lyp2 5′-GTGGAACATCTGAACCAAAG-3′ and 5′-AGCCAAGAGAAATTTTTACCTG-3′; PTPN22.2 5′-CCAAGAGGATGACAGTGTTC-3′ and 5′-CTTTGCTTGACTCCCATCTTTTA-3′; PTPN22.5/6 5′-ATCTGTAATTCTTGCCCACC-3′ and 5′-CTCTTCAGTAAAATAACACACATACC-3′; PTPN22.6 5′-TTTGCCCTATGATTATAGCCG-3′ and 5′-GTTCTCAGGAATTATAAGGACACT-3′; PTPN22.7/8 5′-TCTACAACCCTCCTGGACT-3′ and 5′-TTTCAGCTTCCTTTCCCATT-3′; Ets-1(p51) 5′-CTCCTATGACAGCTTCGACT′3′ and 5′-ATCTCCTGTCCAGCTGATAA-3′; β-actin 5′-GTGACAGCAGTCGGTTGGAG-3′ and 5′-AGGACTGGGCCATTCTCCTT-3′. All real-time PCR reactions were done in duplicate and the transcript levels were normalized against those of β-actin.

### siRNA transfection

One million Jurkat cells were resuspended with 400 ul Opti-mem I containing 400 pmole of siRNA and subjected to electroporation with a Gene Pulser II (Bio-Rad) set at 250 V/400 uF. Human PTPN22 ON-TARGETplus SMARTpool siRNA (L-008066-00-0005), ON-TARGETplus non-targeting siRNA (D-001810-01-05), and siLyp2 (sense: 5′-UGGAAGAAUGGUUCGUUAAUU-3′; anti-sense: 5′-UUAACGAACCAUUCUUCCAUU-3′) were purchased from Dharmacon/Thermo Scientific (Lafayette, CO, USA).

### Statistical analysis

**Figure 1:**
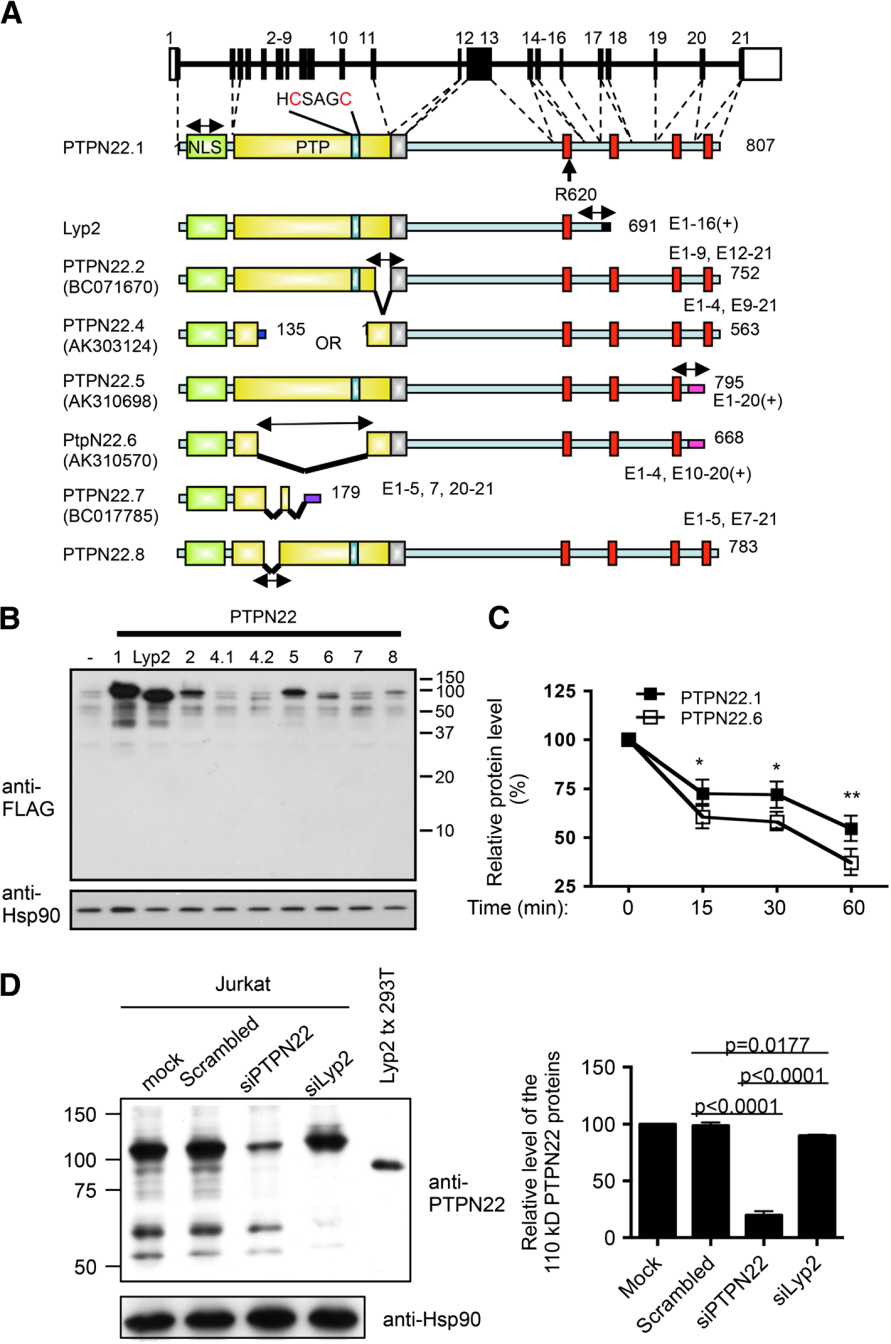
**Alternatively spliced forms of human protein tyrosine phosphatase, non-receptor type 22** (**PTPN22). (A)** Schematic diagrams of the human *ptptn22* gene and putative protein products of each isoform. Exons are numbered, and white boxes represent untranslated exons. Green boxes represent nuclear localization signal (NLS). Yellow boxes represent the protein tyrosine phosphatase (PTP) domains. The key catalytic region (HCSAGC) and R620 are marked. Silver boxes are inhibitory domains. Red boxes represent proline-rich regions. Two-headed arrows indicate the real-time PCR products of the isoforms. The exons constituting each isoform are indicated. The GenBank accession numbers, if available, are listed in parentheses. **(B)** 293 T cells were transfected with 1 μg of an expression vector expressing indicated FLAG-PTPN22 isoforms. The protein levels of FLAG-PTPN22 isoforms and Hsp90 in the transfected cells were determined with Western blotting. **(C)**. 293 T cells were transfected with a plasmid vector expressing FALG-PTPN22.1 and FLAG-PTPN22.6. The transfected cells were treated with 20 ug/ml of cycloheximide. The levels of FLAG-PTPN22.1 and FLAG-PTPN22.6 were then determined with Western blotting and densitometry at indicated time points after the addition of cycloheximide. The level at time 0 for each protein was arbitrarily set as 100%. Statistical significance was determined with paired Student’s *t* test. *P <0.05; **P <0.005. **(D)** Jurkat cells were transfected with indicated siRNA. Cell extract of the transfect cells was then analyzed on Western blotting using anti-PTPN22 and anti-Hsp90 (the left panel). Extract from 293 T cells transfected with an expression vector of Lyp2 was included in the Western blotting (Lyp2 tx 293 T). The levels of the dominant 110 kD PTPN22 protein bands were quantified with a densitometer and normalized against the level of Hsp90 from the corresponding samples, and are shown in the right panel. The normalized level of the mock-transfected cells was arbitrarily set as 100%. Statistical analysis of three independent experiments was performed with one-way ANOVA followed by Tukey’s test.

D’Agostino-Pearson omnibus normality test was used to examine the normality of the data. Statistical analysis was performed with paired Student’s *t* test (Figure 1C), one-way ANOVA (Figures 1D and 3B), the Mann–Whitney test (Figure 4), and Spearman correlation (Figure 5).

## Results

### Identification of additional PTPN22 isoforms

In addition to the published PTPN22 isoforms, we identified several cDNA sequences corresponding to three additional spliced variants of human PTPN22 in the NCBI Gene database (Figure 1A). AK303124 is the product of an out-of-frame splicing between exons 4 and 9. It contains two open reading frames: one of 135 amino acid residues and the other starting at a methionine of exon 9 and corresponding to the last 563 amino acid residues of the full-length PTPN22. AK310698 lacks exon 21 but includes at its C-terminus eight novel amino acid residues encoded by the genomic sequence immediately 3′ to exon 20. BC017785 splices out exons 6 and 8 to 19. We tentatively named these three novel isoforms PTPN22.4, PTPN22.5, and PTPN22.7. We also amplified a novel isoform PTPN22.8 (GenBank accession number JN084012), which lacks exon 6, directly from Jurkat T cells. We were able to confirm the presence of each of the unique or shared spliced junctions in human primary T cells with real-time PCR and DNA sequencing (Figure 1A and data not shown). In addition, we were able to amplify the transcript of each of the isoforms except Lyp2 in its entirety with PCR directly from Jurkat cells. Several attempts to amplify the entire Lyp2 were unsuccessful. The counterpart of PTPN22.2 is also present in rhesus monkeys and chimpanzees, according to NCBI Gene database, suggesting that these alternative splicing events are evolutionarily conserved.

Not all of the isoforms can be expressed efficiently in mammalian cells. We replaced the initiating methionine of each isoform with a FLAG tag and expressed the FLAG-fused PTPN22 proteins in 293 T cells. We found that PTPN22.1 and Lyp2 were expressed more efficiently than PTPN22.2, PTPN22.5, PTPN22.6, and PTPN22.8 (Figure 1B). No protein product of PTPN22.4, either starting from the methionine in exon 1 (4.1) or exon 9 (4.2), or of PTPN22.7 was detected, suggesting that PTPN22.4 and PTPN22.7 are non-productive. We therefore excluded these two isoforms from subsequent functional analyses.

Despite the difference in the protein level, the transcript level of each isoform in transfected cells was very comparable when measured with real-time PCR using a pair of primers targeting the FLAG/PTPN22 fusion junction that is common to all isoforms (data not shown). As the transcripts of these FLAG-PTPN22 proteins contain the same Kozak sequence, initiating ATG, and 3′ polyadenylation tail, which are embedded in the expression vector, we postulated that the difference in the protein level was due to a difference in protein stability instead of translational efficiency. To test this hypothesis, we compared the protein stability between PTPN22.1 and PTPN22.6, which was chosen as an example because it was the least expressed but detectable isoform. We treated transfected cells with cycloheximide to stop protein synthesis and then compared the degradation rate of FLAG-PTPN22.1 and FLAG-PTPN22.6. PTPN22.1 protein was indeed more stable than PTPN22.6 (Figure 1C).

We previously used a PTPN22.6-specific antibody to demonstrate the presence of endogenous PTPN22.6 protein in human T cells. In addition, an anti-human polyclonal PTPN22 antibody, which was raised against S306-S684 of PTPN22.1 and should recognize all isoforms, detected one dominant broad protein band centering around 110 kD and several smaller protein species in Jurkat human T cells but not in HT-29 adenocarcinoma cells, which do not express PTPN22 (Figure 1D and [13]). The levels of almost all of the protein species were reduced by PTPN22 siRNA but not by scrambled siRNA. The commercially available PTPN22 siRNA recognizes four different RNA sequences of PTPN22 and very likely affects the expression of all isoforms. Therefore, the 110 kD broad protein band, which was reduced by approximately 80% by the PTPN22 siRNA, is very likely composed of PTPN22.1, PTPN2.2, PTPN22.5, and PTPN22.8, which have comparable molecular weight. The smaller protein species very likely include Lyp2 and/or yet-to-be-discovered isoforms. However, there is no other isoform-specific antibody, and it is still unclear whether the other non-full-length PTPN22 proteins also exist in human cells. To further confirm their existence, we designed a single siRNA targeting the 3′ end of Lyp2. This siRNA is expected to suppress the expression of Lyp2 and any yet-to-be-discovered isoforms sharing the 3′ end with Lyp2, but not the other isoforms shown in Figure 1A. Indeed, expression of the siLyp2 in Jurkat cells led to the disappearance of the smaller PTP22 protein species including one with a molecular weight comparable to that of Lyp2 (Figure 1D, the left panel), but had a negligible effect on the level of the dominant 110 kD protein band (Figure 1D, the right panel). This result strongly indicates that, in addition to PTPN22.6, endogenous Lyp2 protein is also present in human T cells.

### Expression of PTPN22 isoforms in human PBMC

**Figure 2:**
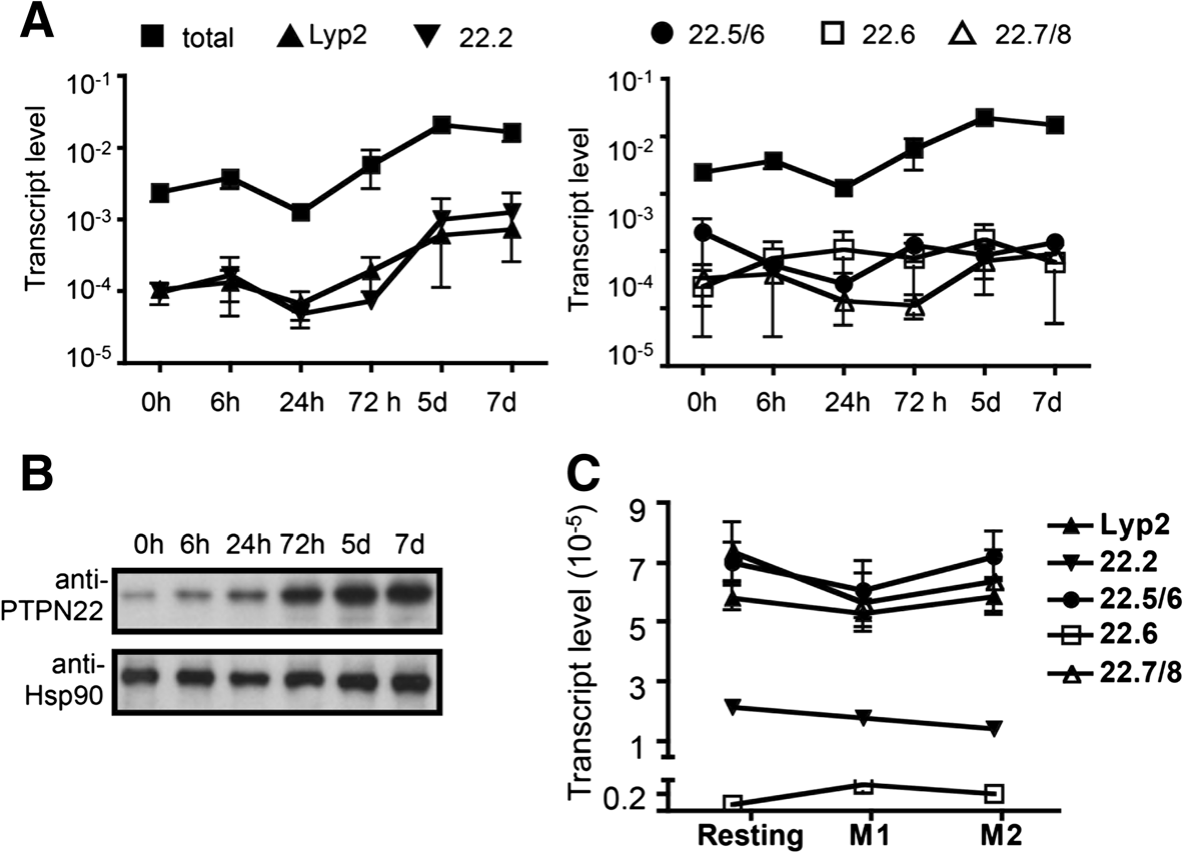
**Expression of protein tyrosine phosphatase, non-receptor type 22 (PTPN22) isoforms in human helper T (Th) cells and macrophages. (A)** RNA was prepared from human Th cells obtained from three healthy donors, which were stimulated *in vitro* with anti-CD3/anti-CD28 for indicated periods of time and subjected to real-time PCR analysis with isoform-specific primers. Each real-time PCR reaction was done in duplicate. The transcript levels thus obtained were normalized against those of β-actin from the same samples. The data shown are the means and standard deviations from three donors. **(B)** Protein extract prepared from human Th cells described in **A** was subjected to Western blotting and probed with PTPN22 antibody and Hsp90 antibody. **(C)** RNA was prepared from resting, M1, and M2 macrophages derived from peripheral blood of seven additional healthy donors and subjected to real-time PCR analysis with isoform-specific primers.

We set out to compare the expression kinetics of each isoform in human T cells. We isolated primary Th cells from the peripheral blood of healthy donors and stimulated the cells with anti-CD3/anti-CD28 for various periods of time. cDNA was prepared from the stimulated cells and subjected to real-time PCR. We designed three pairs of primers specific for PTPN22.2, Lyp2, and PTPN22.6. (Figure 1A). However, PTPN22.1, PTPN22.5, and PTPN22.8 do not have an isoform-specific splice junction. We therefore designed three additional pairs of primers. One pair targeted the 5′ region shared by all isoforms and was used to represent ‘total’ PTPN22 expression. The second pair of primers (PTPN22.5/6) recognized both PTPN22.5 and PTPN22.6 but not other isoforms. The third pair of primers (PTPN22.7/8) was specific to the splice junction shared only by PTPN22.7 and PTPN22.8. We measured the level of PTPN22 isoforms in Th cells obtained from three healthy donors. The data on the level of total PTPN22 and PTPN22.6 were previously reported but were included here for the purpose of comparison. We found that the level of ‘total’ transcript dropped by approximately 50% 24 hours after stimulation and then gradually increased and eventually peaked 5 days after stimulation (Figure 2A). The level of the total PTPN22 transcript was approximately 10 to 50 times more than that of the non-full-length isoforms. The expression kinetics of the non-full-length PTPN22 isoforms can be roughly divided into two groups. The first group including Lyp2 and PTPN22.2 displayed a kinetics similar to that of the total transcript (Figure 2A). In contrast, the transcript level of the second group including PTPN22.5/6, PTPN22.6, and PTPN22.7/8 was not induced throughout the whole course. None of the PTPN22 transcripts were detected at any significant level in HT-29 cells, which expressed no PTPN22 (data not shown). As the transcript levels of the non-full-length isoforms were much lower than that of the total PTPN22 transcript, we believe that the full-length PTPN22 (that is PTPN22.1) is the main contributor to the level of the total PTPN22 transcript. Indeed, the molecular weight of a dominant protein band detected with anti-PTPN22 on Western blotting corresponded to that of PTPN22.1 (Figure 2B). The level of PTPN22.1 protein in primary Th cells also gradually increased after stimulation, a kinetics similar to that of the total transcript of PTPN22 with the exception of no reduction in the levels at 24 hours.

PTPN22 is also expressed in myeloid cells including macrophages. Macrophages can be divided into two major functional subsets: classically activated macrophages (M1 cells) and alternatively activated macrophages (M2 cells). We have recently shown that resting macrophages expressed a basal level of total PTPN22 and that the level stayed relatively unchanged in M1 cells [9]. In contrast, the expression of total PTPN22 was induced in M2 cells to a level three to four times higher than that of resting or M1 cells. To examine the expression of PTPN22 isoforms in macrophages, we quantified the transcript level of PTPN22 isoforms in macrophages from seven healthy donors. We found that the levels of Lyp2, PTPN22.2, PTPN22.5/6, PTPN22.6, and PTPN22.7/8 were very comparable among resting, M1, and M2 macrophages (Figure 2C). Thus, the increase in total PTPN22 observed in M2 cells mainly comes from PTPN22.1. Taken together, our data indicate that the level of PTPN22 isoforms varies significantly among cells types and in response to different stimuli.

### Subcellular localization and function of PTPN22 isoforms

**Figure 3:**
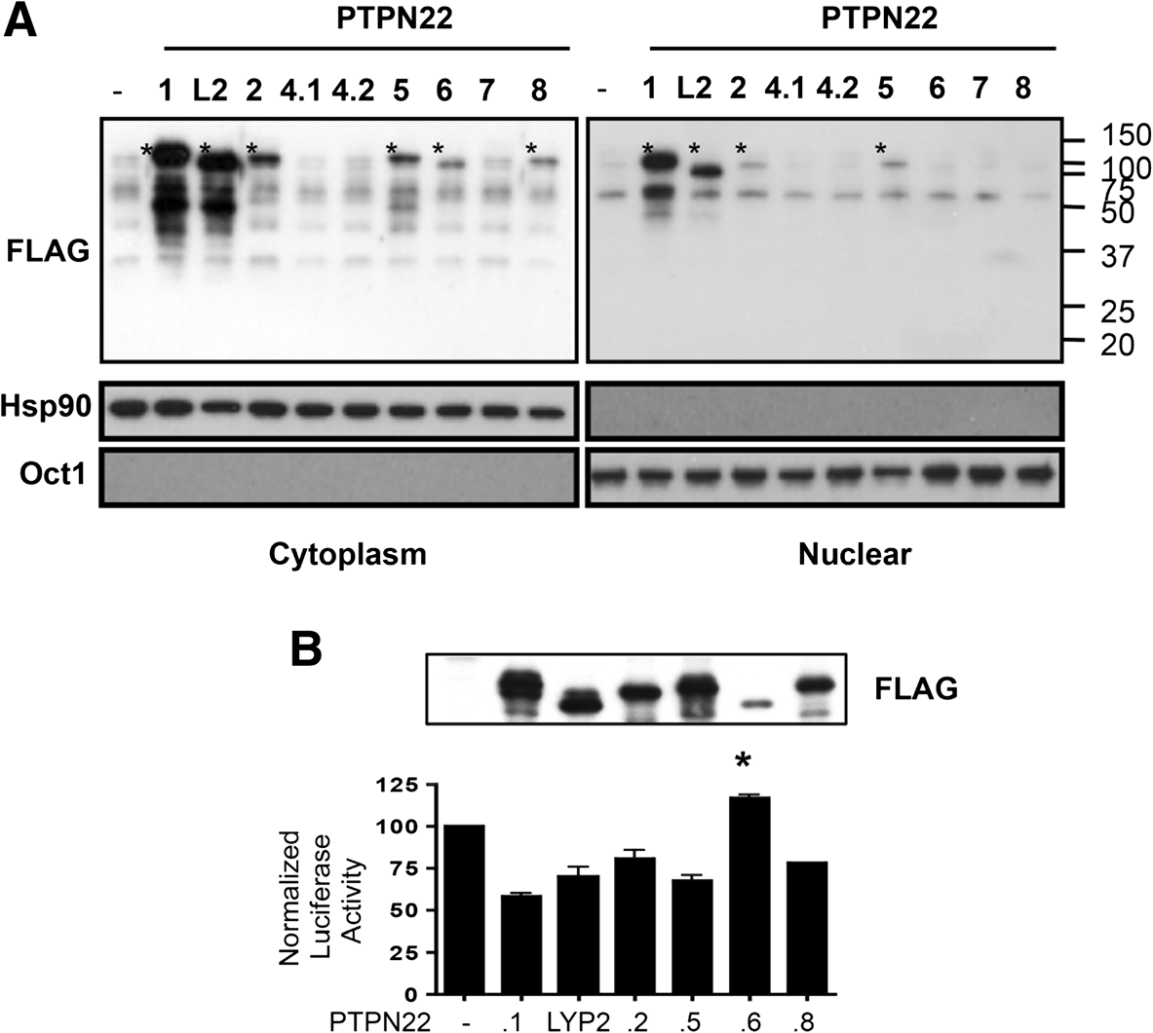
**Subcellular localization and function of protein tyrosine phosphatase, non-receptor type 22 (PTPN22) isoforms. (A)** 293 T cells were transfected with 1 μg of plasmid vectors expressing indicated FLAG-tagged PTPN22 isoforms. Cytoplasmic and nuclear extract was separately prepared from the transfected cells and subjected to Western blotting with indicated antibodies. The protein product of each isoform is marked with ‘*’. **(B)** Jurkat cells were transfected with a NFAT-luc reporter, 10 ug of pCMV2B expressing indicated FLAG-PTPN22 isoforms, and a TK-Renilla reporter. The transfected cells were then stimulated with anti-CD3 overnight. A fraction of the transfected cells was subjected to Western blotting with anti-FLAG (the top panel). The luciferase activity of the remaining cells was analyzed. Normalized firefly luciferase activity was calculated as described in Methods and is shown in the bottom panel. In each experiment, the normalized value from cells transfected with the empty expression vector was arbitrarily set as 100. The data shown are the means and standard deviations of three independent experiments. Statistical significance was calculated with one-way ANOVA followed by Dunn’s test comparing non-full-length isoforms to PTPN22.1. *P = 0.0158.

PTPN22 contains a NLS at its N-terminus and is also present in the nucleus of macrophage and T cells [9]. This NLS is present in all isoforms. To further examine the subcellular localization of PTPN22 isoforms, we expressed each isoform in 293 cells and separately examined the cytoplasmic and nuclear extract of the transfected cells with Western blotting. As expected, PTPN22.1 was detected in both the cytoplasm and the nuclei of the transfected cells (Figure 3A). A similar pattern of subcellular localization was observed for Lyp2, PTPN22.2, and PTPN22.5. Interestingly, we detected PTPN22.6 and PTPN22.8 only in the cytoplasm but not in the nucleus of the transfected cells, suggesting the presence of an additional and essential NLS encoded by exon 6, which is spliced out in these two isoforms.

PTPN22.6 can act as a dominant negative variant of PTPN22.1. But the function of the other isoforms is still unclear. We therefore examined the effect of the other isoforms on NFAT-driven luciferase activity. As expected, overexpression of PTPN22.1 in Jurkat cells suppressed NFAT-dependent luciferase activity by approximately 50%. Interestingly, Lyp2, PTPN22.2, PTPN22.5, and PTPN22.8, despite missing parts of the PTP domain, also had the same effect (Figure 3B). There was no statistically significant difference among these isoforms even after adjustment for the protein level. In addition, a catalytic dead mutant of mouse PTPN22, which contains D195A and C227S mutations [4], had no impact on NFAT activity (Additional file 1), further indicating that these isoforms are still catalytic active. In contrast, expression of PTPN22.6 resulted in a subtle but statistically significant increase in NFAT activity. This result was reported before but was included for comparison [13].

### Expression of PTPN22 isoforms in healthy and SLE populations

To determine whether the expression of PTPN22 isoforms was altered in SLE patients and whether the level of PTPN22 isoforms was correlated with clinical features of SLE, we quantified the transcript level of each isoform in the peripheral blood of 15 healthy donors and 49 patients with SLE. The demographic characteristics of the study subjects are shown in Table 1. All healthy individuals were female, but two of the 49 patients with lupus were male. The average ages were 46.2 years and 48.8 years for the healthy and SLE groups, respectively.

**Table 1:** Demographic characteristics of the study subjects

	**Healthy**	**With systemic lupus erythematosus**
Number and gender	15 F	47 F and 2 M
Age (years)	46.2 ± 5.2	48.8 ± 12.5
Ethnicity	9 Caucasians, 4 blacks, 1 Asians, and 1 Asian/Caucasian	36 Caucasians, 4 blacks, 4 Asians, and 5 Hispanics

**Figure 4:**
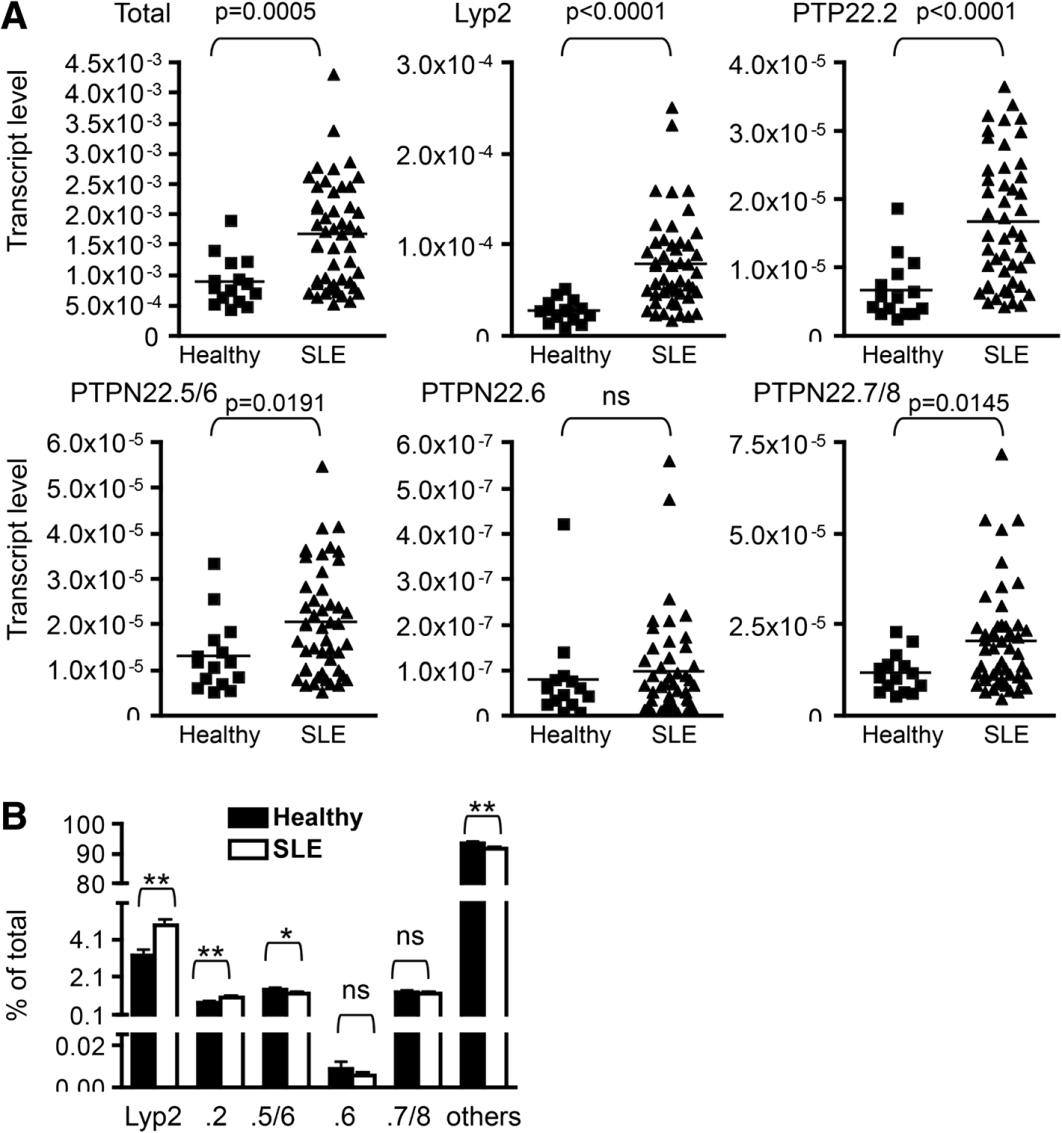
**Expression of protein tyrosine phosphatase, non-receptor type 22 (PTPN22) isoforms in healthy donors and patients with lupus. (A and B)** RNA was prepared from whole blood of healthy individuals (N = 15) and patients with lupus described in Table 1. The RNA was subjected to real-time PCR using PTPN22 isoform-specific primers. The transcript level thus obtained was normalized against that of β-actin obtained from the same sample and is shown in A. The relative level of PTPN22 isoforms is shown in B. Statistical significance was determined with Student’s *t* test. ns stands for not significant. *P <0.05; **P <0.005.

We found that the transcript level of all isoforms except PTPN22.6 was approximately two to three times higher in the SLE group compared to that of the healthy group (Figure 4A). The level of PTPN22.6 was low but comparable in both groups. In addition, the transcript level of Ets-1, a gene that is associated with SLE in Asians [25-27], was also comparable between the two groups (Additional file 2), indicating that the aberrant expression of PTPN22 is not a non-specific event. To determine whether there was preferential expression of any one of the isoforms in SLE patients, we calculated the percentage of each isoform against the total PTPN22 transcript. After deducing the percentage of Lyp2, PTPN22.2, PTPN22.5/6, PTPN22.6, and PTPN22.7/8, the remainder was designated as ‘others’, which we believe was made up of mainly PTPN22.1. We found that SLE patients, compared to healthy individuals, had a modest but statistically significant increase in the percentage of Lyp2 and PTPN22.2 but a reciprocal decrease in the percentage of PTPN22.5/6 and the ‘others’ (Figure 4B). There was no difference in the percentage of PTPN22.6 and PTPN22.7/8. Thus, patients with SLE had not only overexpression of PTPN22 but also an alteration in the relative level of PTPN22 isoforms.

**Figure 5:**
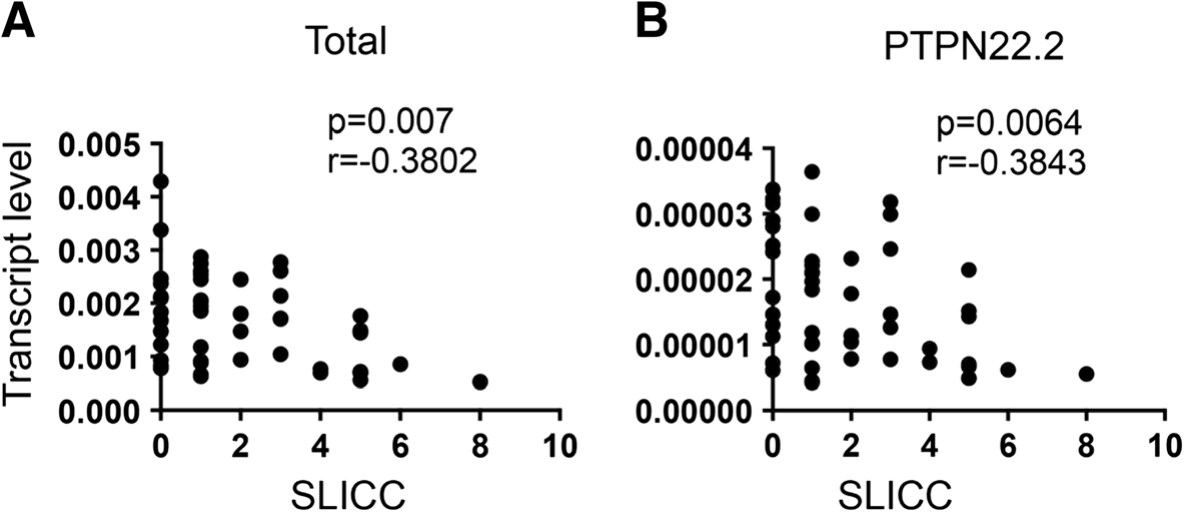
**The level of protein tyrosine phosphatase, non-receptor type 22 (PTPN22) is negatively correlated with the Systemic Lupus International Collaborating Clinics/American College of Rheumatology Damage Indiex (SLICC).** Transcript level of total PTPN22 **(A)** and PTPN22.2 **(B)** in peripheral blood of patients with lupus described in Figure 4 was plotted against SLICC. Correlation was analyzed with a non-parametric Spearman test and adjusted for covariates including gender, age, age of disease onset, duration of disease, and race (white or non-white).

We subsequently examined whether there was any correlation between the transcript level of PTPN22 and clinical parameters of lupus. We found no correlation between total PTPN22 level and SLEDAI, level of antinuclear antibody, or level of anti-double-stranded DNA. However, a significant negative correlation between SLICC-DI and the total transcript level of PTPN22 was observed (Figure 5A). We then examined whether any of the non-full-length isoforms was also negatively correlated with SLICC-DI. We found that the level of PTPN22.2 but not other non-full-length isoforms also showed a negative correlation with SLICSS-DI even after adjustment for covariates including gender, age, age of disease onset, duration of disease, and race (white or non-white) (Figure 5B).

## Discussion

The collection of PTPN22 isoforms has expanded significantly over the past few years. During the preparation of this manuscript, another isoform lacking exon 15 was deposited in the NCBI database as isoform 3 (tentatively called PTPN22.3), which is also present in chimpanzees [28]. The expression of PTPN22.3 in different types of hematopoietic cells and its effect on NFAT activity have yet to be examined. Despite the publication of the cloning of Lyp2, we would like to cautiously point out that the existence of this transcript has yet to be confirmed. The conclusion that this isoform exists is based on the identification of a cDNA fragment corresponding to its unique 3′ end. This isoform was then deduced from several overlapping cDNA fragments [1]. However, there is no full-length cDNA clone in the NCBI database matching Lyp2. In addition, we were unable to amplify the entire transcript of Lyp2 from Jurkat cells. Instead, we recently amplified an intact and novel transcript from Jurkat cells that shares the 3′ end with Lyp2 but contains a deletion in the PTP domain (data not shown). It is very likely that there are other yet-to-be-discovered isoforms sharing the 3′ end with Lyp2. This scenario is consistent with the observation that the siLyp2 suppresses the expression of several PTPN22 protein species (Figure 1D). Ultimately, mass spectrometry and/or isoform-specific antibodies will be needed to confirm the presence of non-full-length PTPN22 proteins in different types of immune cells.

With the exception of PTPN22.6, all isoforms examined in this study are functionally interchangeable in suppressing NFAT activity. However, the function of PTPN22 is still poorly understood. For example, we recently found that cytoplasmic PTPN22 suppresses M1 polarization, whereas nuclear PTPN22 promotes M2 polarization of macrophages [9]. PTPN22 is also expressed in neutrophils and NK cells, and its function in these cells is largely unknown. It is possible that the non-full-length isoforms, each missing a part (or parts) of the PTPN22 protein and some excluded from the nucleus, can also act as dominant negative mutants of PTPN22.1 in other functional readouts or immune cells. Thus, the overall activity of PTPN22 is determined by not only the total level of PTPN22 but also by the functional balance among all isoforms.

The functional balance of PTPN22 isoforms may critically influence the impact of the C1858T SNP. It is still poorly understood how the C1858T SNP increases the risk of SLE and RA but lowers the risk of Crohn’s disease. There are conflicting data on the impact of this SNP on the responsiveness of human T cells to stimulation. We have previously shown that the effect of the C1858T SNP on cytokine production in Th cells is isoform dependent [13]. The R-to-W conversion in the context of PTPN22.1 further weakened NFAT activity and IL-2 production. In contrast, the R-to-W conversion in the context of PTPN22.6 enhanced IL-2 production. If the other non-full-length PTPN22 isoforms also have a function different from that of PTPN22.1, then the cumulative impact of the C1858T SNP can be complicated and highly dependent on the portfolio of PTPN22 isoforms. This scenario may explain the conflicting data described above.

Why do SLE patients express a higher level of PTPN22? The expression of PTPN22 is induced in activated T cells and M2 macrophages. Th and macrophages of patients with lupus are likely activated and express a higher level of PTPN22. The whole blood samples stored in the BWH Lupus Biobank did not allow separate quantification of PTPN22 levels in each type of blood cells. Some types of blood cells, such as T cells, may express more PTPN22 than other types of blood cells, such as macrophages. An expansion of the high PTPN22-expressing cells, but not necessarily an increase in their status of activation, may also contribute to the high level of PTPN22 observed in the whole blood of patients with SLE. Identifying the PTPN22-expressing cells responsible for the higher PTPN22 level in the peripheral blood of patients with SLE may shed light on the pathogenesis of this disease. It is also intriguing to notice that patients with SLE not only have a high level of PTPN22 but also have an altered portfolio of PTPN22 isoforms. They express more Lyp2 and PTPN22.2 at the expense of PTPN22.1 compared to healthy individuals. The clinical significance of such an alteration in the portfolio of PTPN22 isoforms has yet to be determined.

A recent paper by Ronninger *et al*. reported a trend suggesting that the combined transcript level of long PTPN22 isoforms including PTPN22.1, PTPN22.2, and PTPN22.3 was higher in PBMC of patients with RA than healthy controls [29]. Although this trend is consistent with the data shown in Figure 4, their data also showed a trend of lower Lyp2 in patients with RA. In addition, the ratio of long PTPN22 to Lyp2 was significantly higher in patients with RA. We, however, found that the level of Lyp2 was higher while the ratio of long PTPN22 to Lyp2 was lower in our patients with lupus. This discrepancy may originate from fundamental differences in the pathogenesis between RA and lupus. In addition, the primers and probe used to detect long PTPN22 isoforms in Ronninger’s paper target their 3′ end and will not detect PTPN22.5, PTPN22.6, and other yet-to-be-discovered isoforms sharing the 3′ end with PTPN22.5 and PTPN22.6. The difference in primers used may also explain why the ratio of long PTPN22 to Lyp2 is much lower in Ronninger’s study (1 to 1.5) than ours (20 to 30). Our calculated ratio is more consistent with the data shown in Figure 1D.

McKinney *et al*. recently showed that a higher level of PTPN22, along with a low level of ITGA and Notch1 in CD8 + T cells but not PBMC, was associated with a poor prognosis in SLE and anti-neutrophil cytoplasmic antibody (ANCA)-associated vasculitis [30]. The molecular explanation for this correlation is still lacking. The level of PTPN22 in McKinney’s study was indirectly determined by gene chip, which is not specific to any of the PTPN22 isoforms. It will be interesting to know whether such an association could be observed in PBMC if the expression of each of the PTPN22 isoforms was measured. Given the McKinney’s report, it is surprising to find in our study that the level of PTPN22 in peripheral blood was not correlated with SLE disease activity (SLEDAI), but actually was negatively correlated with SLICC-DI. SLICC-DI is usually associated with disease duration. However, we found no correlation between PTPN22 level and disease duration in our SLE population. It is possible that the negative correlation is attributed to damage to one organ. The SLICC-DI in our SLE population was relatively low. However, we did notice a trend suggesting a negative association between PTPN22 levels and damage to the musculoskeletal system (data not shown). More patients will be needed to establish such a negative association.

We do not have a biological explanation for the negative correlation between PTPN22 level and SLICC-DI at this moment. There was a substantial drop in the level of PTPN22 in patients with a SLICC-DI equal to or higher than three. We saw no obvious difference in the portfolio of PTPN22 isoforms in this small group of patients (N = 12) compared to the other patients with SLE. Deficiency of PTPN22 has been shown to lead to hyperactivation of lymphocytes and overexpression of inflammatory cytokines in macrophages [8,9]. Thus, a reduction in the level of PTPN22 as detected in those 12 patients can be proinflammatory, thereby resulting in more organ damage. This hypothesis remains to be confirmed.

## Conclusions

This paper is the first to examine and compare the expression, subcellular localization, and function of various isoforms of PTPN22, a gene that is strongly associated with several rheumatic diseases. Human PTPN22 can be expressed in several isoforms, and some of the isoforms are also present in the nucleus due to at least two essential nuclear localization signals. The expression profile of PTPN22 isoforms varies among cell types, and is altered in patients with lupus. In addition, the levels of total PTPN22 and one of the isoforms are negatively correlated with SLICC-DI scores. Future studies investigating the molecular basis of this negative correlation will provide important insight into the pathogenesis of SLE.

## Abbreviations

cDNA: complementary DNA; DAS28-CRP3: 28-joint disease activity score with three variables including C-reactive protein; DMEM: Dulbecco’s modified Eagle’s medium; IFN: interferon; IL: interleukin; NF-κB: nuclear factor-kappa B; NLS: nuclear localization signal; PBMC: peripheral blood mononuclear cells; PBS: phosphate-buffered saline; PCR: polymerase chain reaction; PTP: protein tyrosine phosphatase; RA: rheumatoid arthritis; SELENA-SLEDAI: safety on estrogens in lupus erythematosus national assessment-SLE disease activity index; siRNA: small inhibitory RNA; SLE: systemic lupus erythematosus; SLICC-DI: Systemic Lupus International Collaborating Clinics/American College of Rheumatology Damage Index; SNP: single nucleotide polymorphism; TCR: T cell receptor; Th: helper T.

## Competing interests

The authors declare that they have no competing interests.

## Authors’ contributions

HHC identified the isoforms of PTPN22, examined their expression, and performed the Western and luciferase assays. WT examined the expression of PTPN22 isoforms in the peripheral blood of healthy donors and patients with lupus. JC performed the statistical analysis of Figure 5. KC recruited patients with lupus and participated in the design and draft of this manuscript. ICH conceived the study and participated in the design and draft of this manuscript. All authors read and approved the final manuscript.

## Supplementary Material

Additional file 1 **Suppression of NFAT activity by protein tyrosine phosphatase, non-receptor type 22 (PTPN22).** Description of data: the data show that a catalytic dead mutant of PTPN22 is not able to suppress NFAT activity. Click here for file

Additional file 2 **Expression of Ets-1 in healthy individuals and patients with systemic lupus erythematosus (SLE).** Description of data: the data show that the expression of Ets-1 is comparable between healthy individuals and patients with SLE. Click here for file
