# Supplementary material for: Altered expression of protein tyrosine phosphatase, non-receptor type 22 isoforms in systemic lupus erythematosus
Source: Arthritis Res Ther. 2014 Jan 17;16(1):R14. doi: 10.1186/ar4440 (PMC3979039; doi:10.1186/ar4440)
Supplement: Additional file 1 — Suppression of NFAT activity by protein tyrosine phosphatase, non-receptor type 22 (PTPN22). Description of data: the data show that a catalytic dead mutant of PTPN22 is not able to suppress NFAT activity. [file ar4440-S1.pdf]

Additional File 1

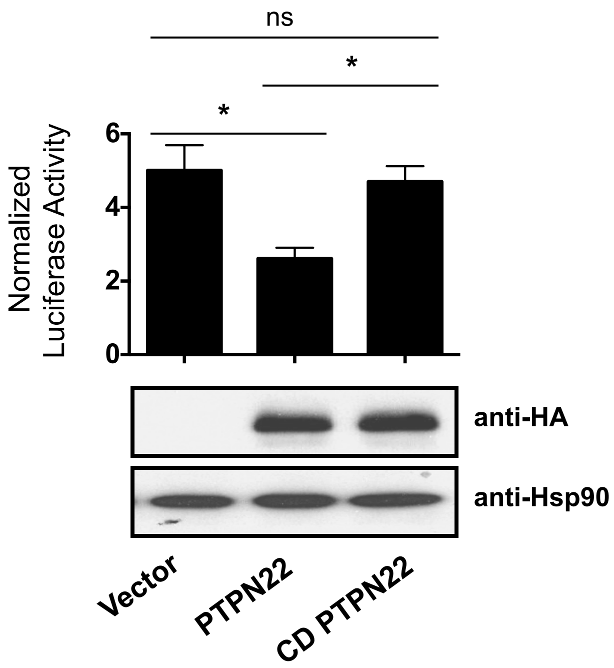

EL4 murine T cells were transfected with a NFAT-luc reporter, 10 ug of pCMV2B expressing indicated HA-tagged mouse PTPN22 or catalytic dead (CD) PTPN22, and a TK-Renilla reporter. The transfected cells were then stimulated with anti-CD3 (1 ug/ml, 145-2C11, Cat. #100331, BioLegend) overnight. A fraction of the transfected cells was subjected to Western blotting with anti-HA (3F10, Cat. #11867423001, Roche) or anti-Hsp90. The luciferase activity of the remaining cells was analyzed. Normalized firefly luciferase activity was calculated as described in *Methods* and is shown. In each experiment, the normalized value from cells transfected with the empty expression vector and un-stimulated was arbitrarily set as 1. The data shown are the means and standard deviations of three independent experiments. Statistical significance was calculated with one-way ANOVA followed by Tukey's test.
