# Supplementary material for: Altered expression of protein tyrosine phosphatase, non-receptor type 22 isoforms in systemic lupus erythematosus
Source: Arthritis Res Ther. 2014 Jan 17;16(1):R14. doi: 10.1186/ar4440 (PMC3979039; doi:10.1186/ar4440)
Supplement: Additional file 2 — Expression of Ets-1 in healthy individuals and patients with systemic lupus erythematosus (SLE). Description of data: the data show that the expression of Ets-1 is comparable between healthy individuals and patients with SLE. [file ar4440-S2.pdf]

Additional File 2

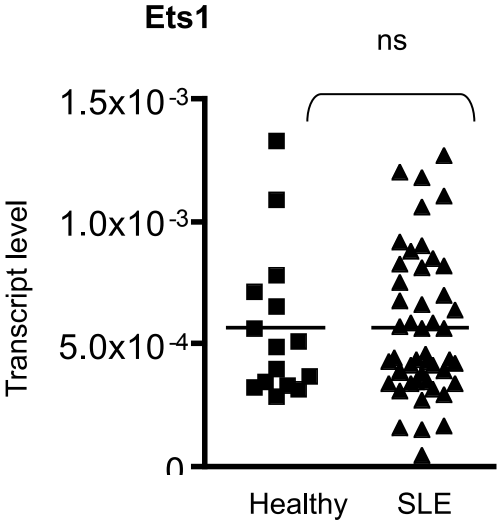

RNA described in Figure 4 was also subjected to real time PCR analysis using Ets-1-specific primers. The transcript level thus obtained was normalized against that of β-actin obtained from the same sample and is shown.
